# Defining Low Milk Supply: A Data-Driven Diagnostic Framework and Risk Factor Analysis for Breastfeeding Women

**DOI:** 10.3390/nu17223524

**Published:** 2025-11-11

**Authors:** Xuehua Jin, Ching Tat Lai, Sharon L. Perrella, Zoya Gridneva, Jacki L. McEachran, Ghulam Mubashar Hassan, Nicolas L. Taylor, Donna T. Geddes

**Affiliations:** 1 School of Molecular Sciences, The University of Western Australia, Crawley, WA 6009, Australia; xuehua.jin@research.uwa.edu.au (X.J.); ching-tat.lai@uwa.edu.au (C.T.L.); sharon.perrella@uwa.edu.au (S.L.P.); zoya.gridneva@uwa.edu.au (Z.G.); jacki.mceachran@uwa.edu.au (J.L.M.); nicolas.taylor@uwa.edu.au (N.L.T.); 2 UWA Centre for Human Lactation Research and Translation, Crawley, WA 6009, Australia; 3 ABREAST Network, Perth, WA 6000, Australia; 4 School of Physics, Mathematics and Computing, The University of Western Australia, Crawley, WA 6009, Australia; ghulam.hassan@uwa.edu.au; 5 ARC Training Centre for Next-Gen Technologies in Biomedical Analysis, The University of Western Australia, Crawley, WA 6009, Australia

**Keywords:** lactation, breastfeeding, milk production, low milk supply, insufficient milk supply, risk factors

## Abstract

**Background:** Current low milk supply (LMS) definitions use subjective maternal perceptions or arbitrary thresholds for 24 h milk production (MP), potentially misclassifying cases. This study aimed to re-evaluate the definition of LMS using data-driven approaches and investigate associated maternal risk factors. **Methods:** Lactating mothers 4–26 weeks postpartum (n = 460) provided demographic, obstetric, and infant data and measured 24 h MP and infant milk intake using the test-weighing method. Infant growth was calculated as their weight-for-age z-score. Latent profile analysis, receiver operating characteristic curve analysis, and multinomial logistic regression were used for classification, diagnostic evaluation, and risk factor assessment for LMS. **Results:** Four milk supply classes emerged: Class 1 with adequate MP, infant intake and infant growth (n = 254); Class 2 with high MP exceeding infant demand and adequate growth (n = 30); Class 3 with slow infant growth despite moderate MP (n = 120); and Class 4 with extremely low MP and high formula intake (n = 56). Classes 1 and 2 were grouped as the normal milk supply group (61.7%), while Classes 3 and 4 formed the LMS group (38.3%). New thresholds were identified for 24 h MP (708 mL/24 h, area under the curve (AUC) = 0.92) and infant breast milk intake (694 mL/24 h, AUC = 0.94) with high diagnostic accuracy. Moreover, practical alternative thresholds for infant average daily weight gain (26 g, AUC = 0.89), formula intake (122 mL/24 h, AUC = 0.89) and formula-to-growth ratio (4 mL/g, AUC = 0.94) were established for the identification of LMS. Minimal breast growth during pregnancy (Odds ratio (OR) = 4.6, 95% confidence interval (CI): 2.3–9.6), advanced maternal age (OR = 2.1, 95% CI: 1.0–4.5), and gestational diabetes mellitus (OR = 2.1, 95% CI: 1.1–4.0) were significant risk factors related to the LMS subgroups. Co-existence of maternal advanced age and overweight showed greatly amplified risk of LMS (OR = 3.7, 95% CI: 1.3–10.5), and a more pronounced risk was observed for the combination of minimal breast growth and advanced maternal age (OR = 9.2, 95% CI: 3.0–28.3). **Conclusions:** This data-driven classification of LMS and identified risk factors may enhance the precision of LMS diagnosis and guide targeted interventions for lactating mothers.

## 1. Introduction

Low milk supply (LMS) is a significant barrier to successful breastfeeding and a primary reason for early formula supplementation and breastfeeding cessation [1]. Accurate identification and management of LMS are therefore critical to supporting lactating mothers and promoting optimal infant nutrition and development. However, current definitions of LMS are limited by a lack of consensus and methodological rigour. Many rely on subjective maternal perceptions or self-reports [2], while objective assessments often adopt arbitrary thresholds for 24 h milk production (MP), such as <600 mL/24 h [3]. These approaches can potentially misclassify cases. Perceived LMS can be inaccurate, influenced by maternal anxiety or expectations [4], and several studies have reported weak correlations between perceived and actual LMS [5,6]. Similarly, fixed thresholds fail to account for individual variability in infant milk requirements and growth trajectories. This imprecision can have important clinical consequences: some mothers may choose unnecessary formula supplementation despite adequate MP, potentially undermining breastfeeding confidence and supply, while others with actual low supply may be overlooked, delaying interventions needed to support infant growth. Such limitations not only hinder clinical decision-making but also constrain research into the underlying causes and consequences of LMS. Increasing evidence suggests that LMS is not a homogenous condition but rather comprises distinct subgroups with varying aetiologies and outcomes [7,8]. A more nuanced, data-driven classification of milk supply could facilitate the development of targeted interventions and improve both diagnosis and care. Furthermore, maternal risk factors such as overweight/obese weight (OW), polycystic ovary syndrome (PCOS) and complications during pregnancy, such as gestational diabetes mellitus (GDM), and birth have been implicated in impaired lactation [8,9,10,11], but the interaction of these factors across different milk supply subgroups requires further investigation. In particular, the co-occurrence of multiple risk factors may amplify breastfeeding difficulties and risk of LMS beyond their individual contributions [12]. Exploring how these factors combine across milk supply subgroups may provide insights into the mechanisms underlying LMS and guide more personalised support strategies.

Therefore, the primary objectives of this study were to (1) identify LMS subgroups using a data-driven clustering approach, (2) re-evaluate and refine the clinical definition of LMS based on empirical evidence, and (3) investigate maternal risk factors associated with these LMS subgroups, including potential compounding effects of associated risk factors. This comprehensive approach was designed to improve our understanding of the diagnosis and management of LMS, thus increasing our potential to improve breastfeeding outcomes for mothers and infants.

## 2. Materials and Methods

### 2.1. Participants

**Figure 1:**
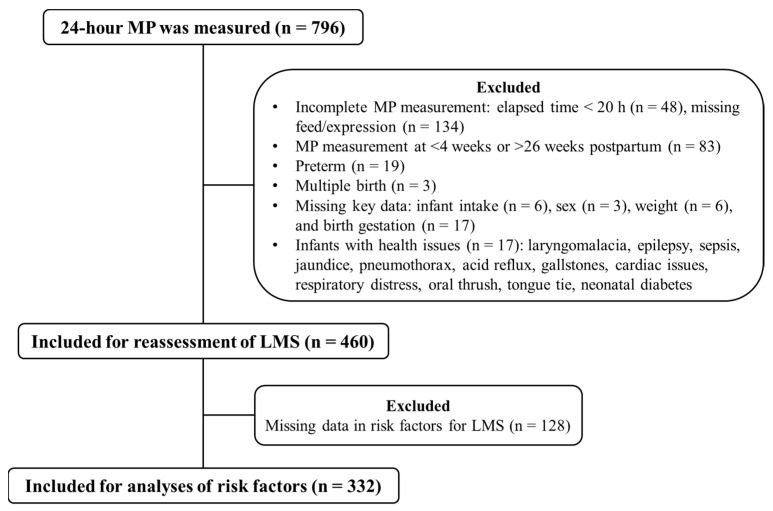
Flow diagram of study participants. LMS: low milk supply; MP: milk production.

This cross-sectional study involved breastfeeding mothers who were recruited through social media from the Perth metropolitan area, Western Australia, and measured their 24 h MP between 2011 and 2025. Breastfeeding mothers were defined as those who breastfed directly or expressed milk either exclusively or occasionally. Eligible participants were English-speaking, fully or partially breastfeeding mothers of singleton infants born at term (birth gestation ≥ 37 weeks) and aged 4–26 weeks. A total of 796 mothers provided 24 h MP records, of whom 460 were retained for further analyses (Figure 1). This study was approved by The University of Western Australia Human Research Ethics Committee (2016055EW RA/4/1/8532, approved on 3 May 2015; 2019/RA/4/20/6134, approved on 21 May 2020). Informed written consent was obtained from all participants.

### 2.2. Data Collection

Given the stability in daily infant intake of HM between 1 and 6 months postpartum [13], 24 h MP measurements were conducted within this temporal range using the test-weighing method [14]. Briefly, the infant was weighed before and after each feed with supplied electronic scales (±2.0 g; Electronic Baby Weigh Scale, Medela Inc., McHenry, IL, USA). Mothers were instructed to record the start and end times for each feed from each breast, and the infant’s weight before and after each breastfeed as well as any expressed milk and commercial milk formula (formula) feeds for 24 h plus one feed. Mothers also recorded the pre and post expression times and weights of any pumping sessions. All milk weights were recorded in grams and expressed in millilitres based on a 1.03 g/mL milk density [15]. Twenty-four-hour MP was calculated with the formula below, where *v_i_* is the volume of each feed/expression, *N* is the total number of feeds and expressions, and *T* is the elapsed time from the end of the first feed until the end of the last feed.

(1) $$MP=\sum_{i=2}^{N}v_{i}\frac{24}{T}$$

Infant 24 h total milk intake was calculated as the sum of all intakes over 24 h, including milk fed directly from the breast and bottle-fed expressed milk or formula.

Mothers completed demographic, obstetric, and infant details questionnaires, including maternal age, height, pre-pregnancy weight, pre-pregnancy and postpartum bra size, education, marital status, perinatal and health complications, parity, birth mode, birth gestation, infant sex, and infant birth weight. Maternal breast volume (BV, cm^3^) for one breast was calculated based on bra cup size and band size, as previously described [16]. Maternal change in BV during pregnancy was calculated as postpartum BV minus pre-pregnancy BV, and minimal breast growth was defined as a change in BV < 100 cm^3^. Maternal BMI was calculated as kg/m^2^, and OW was defined as BMI ≥ 25 kg/m^2^. The infant’s naked weight was measured at home at the time of 24 h MP measurement using Baby Weigh Scales with an accuracy of ±2.0 g (Medela Inc., McHenry, IL, USA). In cases where naked weights were not available (n = 201), infant weight at the first pre-feed weight was adjusted by subtracting 200 g to account for clothing and diaper. Infant weight for age z-score (WAZ) was calculated using the R package ‘anthro 1.0.1’ according to the World Health Organization recommendations [17]. Change in WAZ (∆WAZ) was calculated by subtracting the birth WAZ from the WAZ at the time of 24 h MP measurements. Infant average daily weight gain (g) was calculated by subtracting infant birth weight from the current weight and then dividing by infant age (days). As healthy breastfed newborns typically lose weight during the first few days postpartum and regain their birth weight by around Day 8 [18], we adjusted for this early neonatal period by subtracting 8 days from the infant age when calculating the infant average daily weight gain. The formula-to-growth ratio (mL/g) represents the volume of formula required to achieve one gram of infant weight gain per day and was calculated as infant formula intake (mL) divided by average daily weight gain (g).

### 2.3. Statistical Analysis

Descriptive statistics are presented as mean and standard deviation for continuous variables and frequencies and percentages for categorical variables. Percentages are calculated based on the number of participants with available data for each variable. Differences in continuous variables across multiple groups were assessed using pairwise Wilcoxon rank-sum tests. For categorical variables, pairwise comparisons were conducted using Fisher’s exact test. The significance level was set at *p* < 0.05, and all analyses were carried out in R Statistical Software 4.2.2 (R Foundation for Statistical Computing, Vienna, Austria).

To derive a data-driven and clinically relevant definition of LMS, we applied latent profile analysis (LPA) on five key indicators, including 24 h MP, total infant milk intake, formula intake, WAZ, and ΔWAZ. We used a square root transformation for indicators that were skewed. All indicators were standardised by creating z-scores before LPA. LPA was performed using the R package ‘tidyLPA 1.1.0’. We examined model fit based on our theoretical understanding of the milk supply and the following statistical criteria [19,20]: (1) lower values of Bayesian information criterion (BIC); (2) entropy not less than 0.8, which indicates an acceptable quality of classification and a good indication for class separation; (3) a statistically significant test of the probability that a model with k classes fits better than a model with k − 1 classes using the Bootstrap Likelihood Ratio Test (BLRT); (4) average posterior probabilities of subgroup membership greater than or equal to 0.5 for each subgroup; (5) The smallest class has more than 5% of the individuals in the entire population.

To determine the optimal cut-off value for a dichotomous diagnostic test, Receiver Operating Characteristic (ROC) analysis was conducted on the feeding characteristics and infant weight outcomes using the R package ‘pROC 1.18.2’. In addition to primary indicators, infant average daily weight gain and the formula-to-growth ratio were included due to their practical relevance in clinical settings. These measures can be readily obtained from mothers and serve as feasible alternatives to the more labour-intensive assessment of 24 h MP or infant milk intake.

Multinomial logistic regression was used to assess associations between risk factors and milk supply classes (4-level outcome) using the R package ‘nnet 7.3-18’. An initial multinomial logistic regression model was specified, including ten candidate risk factors and five confounders (infant age at MP measurement [21], sex [22], birth weight [23], birth mode [24], and parity [25]). To identify robust predictors of class membership, we applied a bootstrap resampling approach, repeating the model fitting across 500 bootstrap samples drawn with replacement from the original dataset [26]. All confounders were retained in every model to adjust for potential bias, while variable selection was performed exclusively on the ten risk factors. Risk factors that were selected by more than 50% of bootstrapped models were retained in the final model for interpretation. Results are reported as odds ratios (ORs) with 95% confidence intervals (CI), using Class 1 as the reference. To explore potential compounding associations between risk factors, composite variables were constructed by combining binary predictors hypothesised to occur together (e.g., OW and GDM). These composites were coded as four-level categorical variables (e.g., neither, OW only, GDM only, both) and included in separate multinomial logistic regression models. When assessing combined associations, all confounders and other predictors selected through the earlier bootstrap-based variable selection procedure were retained in the model to ensure appropriate adjustment.

## 3. Results

### 3.1. Participant Characteristics

A summary of maternal, infant and feeding characteristics, and potential risk factors for LMS is presented in Table 1. Complete data were available for 332 (72.2%) participants. The most frequently missing variables were change in BV during pregnancy (n = 74, 16.1%) and maternal BMI (n = 51, 11.1%). Among the ten LMS risk factors assessed, the most common were OW, advanced maternal age, GDM, minimal breast growth during pregnancy, and fertility issues, with prevalence rates ranging from 11.1% to 49.4%.

### 3.2. Reassessment of LMS

**Figure 2:**
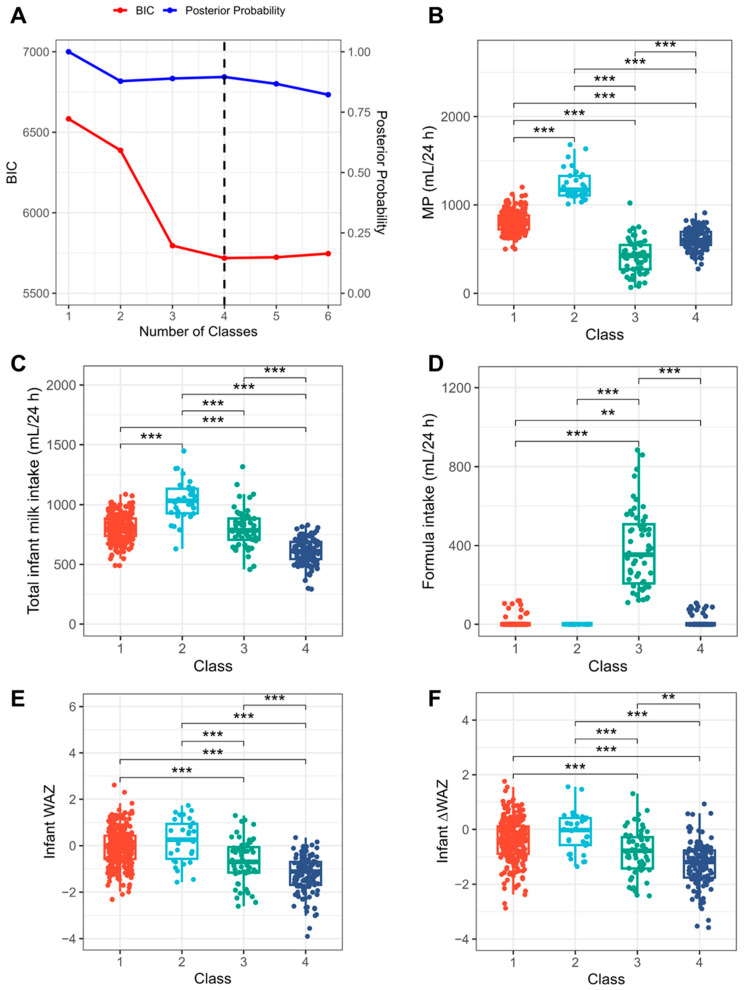
Reassessment of low milk supply (LMS) using latent profile analysis (LPA). (**A**): Model fit assessment for latent profile analysis. The dashed line indicates the number of classes with the best model fit. (**B**–**F**): Distribution of indicator variables across the identified latent classes. **: *p* < 0.01; ***: *p* < 0.001. BIC: Bayesian information criterion; MP: milk production; WAZ: weight-for-age z-score; ΔWAZ: change in weight-for-age z-score from birth.

Subgroups of milk supply were identified using LPA model fit assessment (Figure 2A, Supplementary Table S1). The best mode fit selected was a four-class solution that had a low BIC (5718.6), a high entropy value (0.821), and a high average posterior probability (0.896). Class 1 (n = 254) was characterised with adequate MP (median = 789 mL/24 h), infant intake (median = 801 mL/24 h) and growth (WAZ median = −0.05); Class 2 (n = 30) with high MP (median = 1171 mL/24 h) exceeding infant demand (median = 1032 mL/24 h) and adequate growth (WAZ median = 0.26); Class 3 (n = 56) with extremely low MP (median = 427 mL/24 h) and high formula intake (median = 354 mL/24 h); and Class 4 (n = 120) with slow infant growth (WAZ median = −1.11) despite moderate MP (median = 604 mL/24 h) (Figure 2B–F).

**Figure 3:**
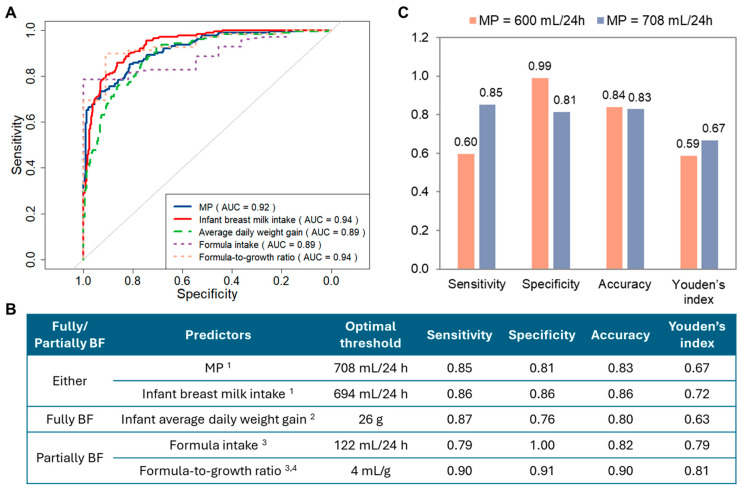
Receiver operating characteristic (ROC) analysis of predictors of low milk supply (LMS). (**A**): ROC curves for predictors of LMS. (**B**): Performance metrics and optimal thresholds for individual LMS predictors. ^1^ ‘MP’ and ‘Infant breast milk intake’ were analysed using the full dataset (n = 460). ^2^ ‘Infant average daily weight gain’ was analysed using the full dataset (n = 460) and reflects weight gain attributed to breast milk intake; ^3^ ‘Formula intake’ and ‘Formula-to-growth ratio’ were analysed only among partially breastfeeding dyads (n = 81); ^4^ ‘Formula-to-growth ratio’ represents the volume of formula (mL) required to achieve one gram of infant weight gain per day. (**C**): Comparison of previously proposed and newly identified thresholds of milk production (MP) for predicting LMS. AUC: area under the curve; BF: breastfeeding.

Classes 1 and 2 were grouped as the NMS group (n = 284, 61.7%), while Classes 3 and 4 were grouped as the LMS group (n = 176, 38.3%). ROC curve analysis was conducted to assess the ability of potential predictors to discriminate between LMS and NMS (Figure 3A,B). In research settings where detailed measurement is feasible, 24 h MP and infant breast milk intake were strong predictors of LMS, with optimal cut-offs of 708 mL/24 h and 695 mL/24 h, respectively. In clinical settings, infant average daily weight gain emerged as a practical predictor among fully breastfeeding dyads, with infants gaining less than 26 g/day classified as LMS. Among partially breastfeeding dyads, formula intake exceeding 122 mL/24 h and formula-to-growth ratio higher than 4 mL/g were strong predictors of LMS.

Figure 3C compares prediction metrics between the previously proposed MP threshold (600 mL/24 h) and the newly identified MP threshold (708 mL/24 h) for predicting LMS. The new threshold improved sensitivity and Youden’s index, while the old threshold offered higher specificity.

### 3.3. Risk Factors for LMS

Of the ten assessed risk factors, participants had up to six concurrently (Supplementary Table S2). Among all groups, Class 2 had the highest proportion of mothers with no identified risk factors (31.8%). In contrast, Class 1 showed the highest proportion of participants with one risk factor (27.8%), while Class 2 showed the highest proportion with two risk factors (30.3%). Higher cumulative risk burden (≥3 risk factors) was more prevalent in Class 3, which shows an increasing burden of risk factors in the LMS groups, particularly in Class 3, compared to the NMS groups.

The prevalence of risk factors for LMS varied across the four milk supply classes (Table 2). Maternal OW was most common in Class 3 (66.0%), with a significantly higher prevalence than in Class 2 (18.2%). Advanced maternal age was also most frequent in Class 3 (51.1%), which differed significantly from Class 1 (32.1%) and Class 4 (25.0%). GDM occurred most often in Class 4 (32.9%), with a prevalence significantly higher than in Class 1 (19.8%). Minimal breast growth during pregnancy was most frequent in Class 3 (51.1%), significantly exceeding the prevalence in Class 1 (19.3%) and Class 4 (17.1%). Other risk factors, including fertility issues, thyroid disorders, PCOS, nipple piercing or breast surgery, postpartum haemorrhage, and hypertensive disorders in pregnancy, showed relatively low prevalence (<15%) and did not differ significantly between classes.

**Figure 4:**
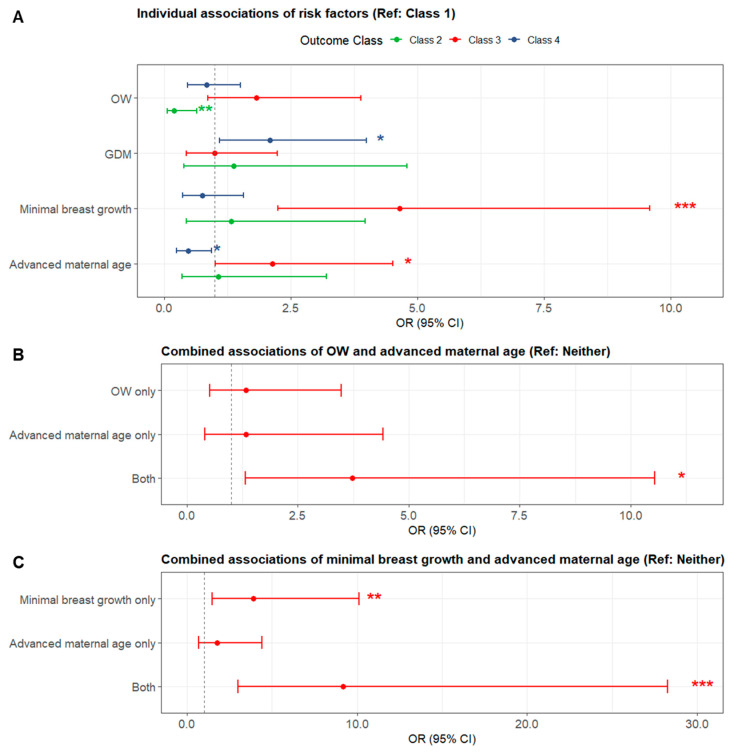
Forest plots of risk factors for low milk supply. (**A**): Individual effects; (**B**): Compounding effect of OW and advanced maternal age (Class 3 vs. Class 1); (**C**): Compounding effect of minimal breast growth and advanced maternal age (Class 3 vs. Class 1). CI: confidence interval; GDM: gestational diabetes mellitus; OR: odds ratio; OW: overweight/obese weight; *: *p* < 0.05; **: *p* < 0.01; ***: *p* < 0.001.

Multivariable logistic regression identified several significant associations with milk supply subgroups (Figure 4A and Supplementary Table S3). Compared to Class 1, mothers in Class 2 had a significantly lower likelihood of being OW (OR = 0.197, 95% CI: 0.061–0.642, *p* = 0.007), with no other predictors reaching significance. In Class 3, minimal breast growth during pregnancy was strongly associated with increased risk (OR = 4.646, 95% CI: 2.251–9.590, *p* < 0.001), and advanced maternal age was also significant (OR = 2.142, 95% CI: 1.016–4.517, *p* = 0.045). In Class 4, GDM (OR = 2.096, 95% CI: 1.099–3.997, *p* = 0.025) and younger maternal age (OR = 0.481, 95% CI: 0.248–0.933, *p* = 0.030) were significantly associated with class membership.

Concurrent OW and advanced maternal age demonstrated a greater membership of Class 3 (Figure 4B and Supplementary Table S4). While neither factor alone significantly increased the odds of being Class 3 compared to Class 1, co-existence of these risk factors resulted in 3.7-fold higher odds (OR = 3.728, 95% CI: 1.319–10.538, *p* = 0.013). An even more pronounced risk was observed for the combination of minimal breast growth and advanced maternal age (Figure 4C and Supplementary Table S4). Minimal breast growth alone was associated with 3.9-fold increased odds of Class 3 (OR = 3.892, 95% CI: 1.499–10.104, *p* = 0.005), while advanced maternal age alone was not significant (OR = 1.760, 95% CI: 0.700–4.424, *p* = 0.229). However, when both factors were present, the odds increased to 9.2-fold (OR = 9.199, 95% CI: 2.995–28.253, *p* < 0.001).

## 4. Discussion

In this study, we applied LPA to classify adequacy of milk supply into distinct subgroups based on maternal MP, infant intake, and infant growth outcomes. The best-fitting solution identified four classes, with Class 3 reflecting severe LMS. Mothers in this group had extremely low MP and relied heavily on formula supplementation. This suggests that they were aware of low supply issues and proactively started supplementation to achieve or maintain adequate infant growth. Class 4 presents a more subtle and concerning situation where most mothers had MP (median = 604 mL/24 h) that appeared sufficient by a previously proposed definition, yet their infants showed slow growth and received little formula supplementation, suggesting inadequate total milk intake. Previous studies have shown that mothers frequently underestimate their milk supply, leading to early weaning or unnecessary formula supplementation [27,28]. However, our findings suggest that mothers in Class 4 experienced delayed recognition or response to low supply. These mothers require particular attention and support to ensure timely intervention and prevention of infant undernutrition. While our primary focus was on identifying LMS, the data-driven approach also suggested the existence of a potential oversupply group (Class 2, 6.5%), where MP exceeded infant demand. Oversupply has received little attention in the literature and also lacks a clear definition. Some mothers describe oversupply positively, reporting feelings of reward and willingness to donate milk [29,30], yet others experience adverse outcomes such as infant feeding difficulties or maternal discomfort [31]. Importantly, perceived oversupply may sometimes reflect ineffective milk transfer or a strong milk ejection reflex rather than actual excess production [32]. Similar to LMS, oversupply should therefore be considered in relation to MP, infant demand, and growth outcomes, rather than perception alone.

We identified an optimal cut-off of 708 mL/24 h for MP to flag LMS. This value is higher than the previously proposed 600 mL/24 h threshold. The new cut-off improved sensitivity and showed a higher Youden’s index, meaning it performed better overall in distinguishing LMS from NMS. Greater sensitivity may lead to more false positives, but this is unlikely to cause harm. Mothers who are flagged at risk would be further evaluated and typically offered extra breastfeeding support, which is helpful regardless of their final classification [33]. On the other hand, false negatives pose a much greater concern, as missed cases of LMS can delay recognition and compromise infant nutrition and growth. We also identified practical alternatives when 24 h MP or intake measurements are not feasible. For fully breastfeeding dyads, infant average daily weight gain below 26 g/day indicated LMS, which is 30% higher than the cut-off of 20 g/day weight velocity used in a previous study [34]. For breastfeeding mothers who also feed formula due to choice or practical challenges such as returning to work, working night shifts, or other life demands [35], cut-offs for formula intake and the formula-to-growth ratio can provide reliable markers of supply adequacy. These measures not only support research and clinical practice but may also help mothers self-assess their supply and decide if additional support is needed. Although MP, infant intake, and weight gain are strong individual predictors, relying on a single measure can still lead to misclassification. A combined, multi-indicator approach that includes consideration of maternal and infant health and patterns of weight gain over time may therefore provide more accurate identification of LMS in real-world clinical practice.

Several maternal risk factors were associated with LMS in this study. The association with GDM is well supported by previous work, showing that GDM may delay secretory activation and increase the likelihood of LMS [36,37,38]. Minimal breast growth during pregnancy was another strong indicator. Together with wide intermammary spacing, minimal or no breast growth during pregnancy may be associated with breast hypoplasia, which is also linked to LMS [39,40]. A recent study using ultrasound imaging to assess breast anatomy also suggested that a minimal breast growth was correlated with reduced mammary blood flow and fewer main milk ducts with smaller diameters, which in turn correlated with a lower MP [25]. Advanced maternal age also appeared to play a role. Older mothers face higher risks of metabolic disorders and pregnancy complications including hypertension, GDM, and postpartum haemorrhage [41]. These conditions themselves are risk factors for impaired lactation [42]. The accumulation of such age-related conditions may explain the greater burden of risk that we observed in LMS groups.

Our results also suggest that when there are co-existing maternal risk factors, these result in a substantially higher likelihood of LMS. Several biological mechanisms potentially explain these interactions. Both maternal OW and advanced age are associated with reduced insulin sensitivity, which may compromise the insulin signalling required for mammary secretory differentiation [43,44]. Minimal breast growth during pregnancy may reflect reduced development of glandular tissue, which cannot respond fully to hormonal signals [45,46]. Minimal breast growth may also be linked to PCOS, as common features such as insulin resistance, OW, hyperandrogenism, and low progesterone levels, may contribute to insufficient glandular tissue development [47]. When such risks overlap, the cumulative physiological burden may exceed the body’s adaptive capacity, leading to more severe LMS. Most studies on breastfeeding challenges have focused on individual risk factors, and only a few have looked at how they interact. One study showed that the combination of maternal OW and GDM was associated with significantly lower infant milk intake at 3 months postpartum [48]. Another reported that the same combination more than doubled the risk of not fully breastfeeding at 6 to 8 weeks postpartum [12]. Our findings are also consistent with broader evidence in perinatal health, where combinations of maternal risk factors amplify the chances of adverse infant outcomes [49,50]. However, no studies to date have investigated how combined maternal risk factors relate to LMS, highlighting the need for future research to validate the present findings. Clinically, these risk factors could be incorporated into routine antenatal assessments through structured checklists alongside standard obstetric evaluations. Mothers identified as high risk could benefit from early lactation consultations, MP measurement, and close monitoring of infant weight gain.

This study has several strengths. For the first time, LMS was assessed using a data-driven approach that integrated maternal MP, infant intake, and growth outcomes. This comprehensive framework helps overcome the shortcomings of previous definitions that relied on perception or arbitrary cut-offs. In addition, by examining multiple maternal risk factors in combination rather than isolation, we were able to identify critical compounding effects that are often overlooked in traditional analyses. At the same time, some limitations should be acknowledged. The prevalence of LMS identified in this study (38.3%) may be higher than in the general population, as women who had breastfeeding problems were more likely to participate in our study to seek information and support. Moreover, cut-offs for formula intake and formula-to-growth ratio were analysed only among partially breastfeeding dyads (n = 81), where the sample size was relatively small compared to the whole study cohort. Lastly, approximately half of the examined risk factors were relatively rare (<10%), which might have limited the statistical power to detect independent associations and may explain why some were not retained in regression models. However, the lack of statistical significance does not diminish their potential importance. As the cumulative number of such risk factors increases, their combined effect may still be clinically meaningful. Future research with larger and more diverse cohorts will be essential to validate these findings and further refine risk profiles for LMS.

## 5. Conclusions

In conclusion, this study identified meaningful subgroups of milk supply using a data-driven approach, moving beyond definitions based solely on perception or arbitrary cut-offs. By refining the diagnostic threshold and introducing practical alternatives, we provide tools that may enhance clinical identification of LMS. Notably, the compounding effects of multiple maternal risk factors highlight the need for comprehensive lactation risk assessment during the perinatal period. Together, these insights offer a foundation for more accurate diagnosis of LMS, more personalised support for mothers, with the potential to improve breastfeeding outcomes and infant growth.

## Figures and Tables

**Table 1:** Participant characteristics.

	Value ^1^	Missing ^2^
**Maternal characteristics**		
Age (years)	33.1 ± 4.3 (22.7–45.9)	4 (0.9%)
Change in BV during pregnancy (cm^3^)	185 ± 134 (−230–660)	74 (16.1%)
BMI (kg/m^2^)	26.2 ± 5.7 (17.2–62.5)	51 (11.1%)
Education: bachelor or above	331 (72.1%)	1 (0.2%)
Marital status: married or de facto	438 (95.4%)	1 (0.2%)
Parity: primiparous	273 (59.3%)	0
Birth mode: vaginal	267 (60.3%)	17 (3.7%)
**Infant characteristics**		
Sex: male	207 (45%)	0
Birth gestation (weeks)	39.3 ± 1.1 (37.0–42.4)	0
Birth weight (g)	3.4 ± 0.4 (2.0–5.0)	0
Birth WAZ	0.2 ± 0.9 (−3.2–3.5)	0
WAZ at MP measurement	−0.4 ± 1.0 (−3.9–2.6)	0
∆WAZ	−0.7 ± 0.9 (−3.6–1.8)	0
Average daily weight gain (g)	29.0 ± 9.0 (−1.0–63.1)	0
Age at MP measurement (weeks)	12.8 ± 5.1 (4.2–25.9)	0
**Feeding characteristics**		
Fully breastfeeding	379 (82.4%)	0
MP (mL/24 h)	734 ± 228 (67–1682)	0
Total infant milk intake (g/24 h) ^3^	771 ± 162 (293–1448)	0
Infant breast milk intake (g/24 h)	721 ± 202 (39–1448)	0
Infant formula intake (g/24 h)	51 ± 142 (0–884)	0
Breastfeeding duration (min/feed)	13.2 ± 7.0 (0–45)	0
Total milk removal frequency (times/24 h) ^4^	13.6 ± 4.4 (6–31)	0
Breastfeeding frequency (times/24 h) ^5^	11.0 ± 4.6 (0–29)	0
Breast expression frequency (times/24 h) ^5^	2.6 ± 4.0 (0–20)	0
**Risk factors for LMS**		
OW (BMI ≥ 25 kg/m^2^)	202 (49.4%)	51 (11.1%)
Advanced maternal age (≥35 years)	141 (30.9%)	4 (0.9%)
GDM	103 (22.4%)	0
Minimal breast growth during pregnancy (<100 cm^3^)	92 (23.8%)	74 (16.1%)
Fertility issues	51 (11.1%)	0
Thyroid disorders	41 (8.9%)	0
PCOS	40 (8.7%)	0
Nipple piercing or breast surgery	35 (7.6%)	0
Postpartum haemorrhage	29 (6.3%)	0
Hypertensive disorders in pregnancy	23 (5.0%)	0

^1^ Values are mean ± SD (range) or n (%), and percentages are calculated based on the number of participants with available data; ^2^ Percentages are calculated based on the total number of participants (n = 460); ^3^ Total infant milk intake is sum of infant breast milk intake and commercial milk formula intake; ^4^ Total milk removal frequency is the combined frequencies of breastfeeding and breast expression; ^5^ Breastfeeding and expression frequencies were calculated per breast; BMI: body mass index; BV: breast volume; GDM: gestational diabetes mellitus; LMS: low milk supply; MP: milk production; OW: overweight/obesity; PCOS: polycystic ovary syndrome; WAZ: weight-for-age z-score.

**Table 2:** Prevalence of risk factors for low milk supply among different classes.

Risk Factors for LMS	Class 1 (n = 187)	Class 2 (n = 22)	Class 3 (n = 47)	Class 4 (n = 76)
OW (BMI ≥ 25 kg/m^2^)	101 (54.0%) a	4 (18.2%) b	**31 (66.0%)** ac	37 (48.7%) ac
Advanced maternal age (≥35 years)	60 (32.1%) a	6 (27.3%) ab	**24 (51.1%)** b	19 (25.0%) a
GDM	37 (19.8%) a	4 (18.2%) ab	14 (29.8%) ab	**25 (32.9%)** b
Minimal breast growth during pregnancy (<100 cm^3^)	36 (19.3%) a	6 (27.3%) ab	**24 (51.1%)** b	13 (17.1%) a
Fertility issues	20 (10.7%)	3 (13.6%)	**7 (14.9%)**	11 (14.5%)
Thyroid disorders	17 (9.1%)	**3 (13.6%)**	4 (8.5%)	6 (7.9%)
PCOS	17 (9.1%)	**3 (13.6%)**	4 (8.5%)	7 (9.2%)
Nipple piercing or breast surgery	**15 (8** **.0** **%)**	0 (0%)	2 (4.3%)	5 (6.6%)
Postpartum haemorrhage	16 (8.6%)	2 (9.1%)	**5 (10.6%)**	4 (5.3%)
Hypertensive disorders in pregnancy	**16 (8.6%)**	0 (0%)	1 (2.1%)	2 (2.6%)

Results are presented as n (%), with percentages calculated based on the sample size of each class; Bold indicates the subclass with the highest prevalence of each risk factor; Subclasses not sharing the same letter for each risk factor differ significantly (*p* < 0.05), as determined by pairwise Fisher’s exact tests; BMI: body mass index; GDM: gestational diabetes mellitus; LMS: low milk supply; OW: overweight/obesity; PCOS: polycystic ovary syndrome.

## Data Availability

Restrictions apply to the availability of some or all data generated or analysed during this study. The corresponding author will, on request, detail the restrictions and any conditions under which access to some data may be provided.

## Supplementary Materials

The following supporting information can be downloaded at: https://www.mdpi.com/article/10.3390/nu17223524/s1, Table S1: Latent profile analysis model fit assessment; Table S2: Number of risk factors for low milk supply among different classes; Table S3: Multinomial logistic regression on individual risk factors; Table S4: Combined associations of composite risk factors.

## Abbreviations

The following abbreviations are used in this manuscript:

BIC	Bayesian information criterion
BLRT	Bootstrap likelihood ratio test
BMI	Body mass index
BV	Breast volume
CI	Confidence interval
GDM	Gestational diabetes mellitus
LPA	Latent profile analysis
LMS	Low milk supply
MP	Milk production
NMS	Normal milk supply
OW	Overweight/obese weight
PCOS	Polycystic ovary syndrome
ROC	Receiver operation characteristics
OR	Odds ratio
WAZ	Weight-for-age z-score
