# Supplementary material for: Defining Low Milk Supply: A Data-Driven Diagnostic Framework and Risk Factor Analysis for Breastfeeding Women
Source: Nutrients. 2025 Nov 11;17(22):3524. doi: 10.3390/nu17223524 (PMC12655712; doi:10.3390/nu17223524)
Supplement: Supplementary file 1 [file nutrients-17-03524-s001.zip › nutrients-3969465-supplementary.pdf]

Defining Low Milk Supply: A Data-Driven Diagnostic Framework and Risk Factor Analysis for Breastfeeding Women

Xuehua Jin[1,2,3], Ching Tat Lai[1,2,3], Sharon L. Perrella[1,2,3], Zoya Gridneva[1,2,3], Jacki L. McEachran[1,2,3], Ghulam Mubashar Hassan[4], Nicolas L. Taylor[1,5], and Donna T. Geddes[1,2,3,5,*]

SUPPLEMENTARY MATERIALS

Table S1. Latent profile analysis model fit assessment

Classes	BIC	SABIC	Entropy	$P_{average}$	N_{min}	*p*BLRT
1	6583.4	6551.7	1	1	1	NA
2	6387.4	6336.7	0.601	0.878	0.460	0.010
3	5795.9	5726.1	0.772	0.889	0.128	0.010
**4**	**5718.6**	**5629.7**	**0.821**	**0.896**	**0.065**	**0.010**
5	5723.7	5615.8	0.794	0.867	0.067	0.010
6	5746.4	5619.5	0.748	0.822	0.065	0.109

Bold indicates the best model fit. BIC: Bayesian information criterion; N_{min}: the proportion of whole population the smallest class has; $P_{average}$: the smallest average posterior probabilities of subgroup membership; *p*BLRT: bootstrap likelihood ratio test *p*-value; SABIC: sample size-adjusted BIC.

Table S2. Number of risk factors for low milk supply among different classes

Number of risk factors	Class 1	Class 2	Class 3	Class 4
0	14.4%	31.8% [1,2]	6.4%	17.1%
1	27.8% [1,2]	18.2%	19.1%	25.0%
2	25.7%	27.3%	27.7% [1]	30.3% [1,2]
3	21.9%	18.2%	23.4% [2]	19.7%
4	7.0%	4.5%	12.8% [2]	6.6%
5	2.7%	0	8.5% [2]	1.3%
6	0.5%	0	2.1% [2]	0

[1] The highest proportion in each column; [2] The highest proportion in each row.

Table S3. Multinomial logistic regression on individual risk factors

Ref: Class 1	Predictors/confounders	OR	Lower CI	Upper CI	*p*-value
Class 2	OW (yes vs. no)	0.197	0.061	0.642	0.007

	Advanced maternal age (yes vs. no)	1.070	0.357	3.208	0.904
	GDM (yes vs. no)	1.375	0.394	4.796	0.618
	Minimal breast growth during pregnancy (yes vs. no)	1.324	0.442	3.966	0.616
	Hypertensive disorders in pregnancy (yes vs. no)	-	-	-	-
	Infant birth weight	1.114	0.395	3.141	0.838
	Infant age at MP measurement	1.159	1.053	1.276	0.002
	Male vs. Female	1.607	0.596	4.336	0.349
	Primiparous vs. Multiparous	1.873	0.630	5.573	0.259
	C-section vs. Vaginal birth	1.029	0.359	2.953	0.957
Class 3	OW (yes vs. no)	1.828	0.859	3.889	0.117
	Advanced maternal age (yes vs. no)	2.142	1.016	4.517	0.045
	GDM (yes vs. no)	0.997	0.444	2.236	0.994
	Minimal breast growth during pregnancy (yes vs. no)	4.646	2.251	9.590	<0.001
	Hypertensive disorders in pregnancy (yes vs. no)	0.255	0.032	2.059	0.200
	Infant birth weight	0.576	0.257	1.292	0.181
	Infant age at MP measurement	0.990	0.923	1.062	0.785
	Male vs. Female	0.952	0.470	1.929	0.891
	Primiparous vs. Multiparous	1.290	0.597	2.783	0.517
	C-section vs. Vaginal birth	1.873	0.906	3.871	0.090
Class 4	OW (yes vs. no)	0.839	0.465	1.513	0.560
	Advanced maternal age (yes vs. no)	0.481	0.248	0.933	0.030
	GDM (yes vs. no)	2.096	1.099	3.997	0.025
	Minimal breast growth during pregnancy (yes vs. no)	0.760	0.368	1.573	0.460
	Hypertensive disorders in pregnancy (yes vs. no)	0.277	0.058	1.320	0.107
	Infant birth weight	0.508	0.268	0.965	0.038
	Infant age at MP measurement	1.010	0.954	1.069	0.731
	Male vs. Female	0.636	0.357	1.134	0.125
	Primiparous vs. Multiparous	0.490	0.273	0.881	0.017
	C-section vs. Vaginal birth	1.640	0.907	2.967	0.102

-: No hypertensive disorders were reported in Class 2; CI: confidence interval; GDM: gestational diabetes mellitus; MP: milk production; OR: odds ratio; OW: overweight/obese weight.

Table S4. Combined effects of composite risk factors

Ref: Class 1	Predictors (ref: neither)	OR	Lower CI	Upper CI	*p*-value
Class 2	OW only	0.050	0.006	0.396	0.005
	GDM only	0.275	0.032	2.341	0.237
	Both OW and GDM	0.925	0.218	3.928	0.916
Class 3	OW only	1.145	0.490	2.675	0.755
	GDM only	0.249	0.048	1.299	0.099
	Both OW and GDM	2.319	0.816	6.588	0.114
Class 4	OW only	0.765	0.384	1.523	0.445
	GDM only	1.620	0.663	3.957	0.290
	Both OW and GDM	2.040	0.825	5.046	0.123
Class 2	OW only	0.185	0.048	0.721	0.015
	Minimal breast growth only	1.372	0.397	4.745	0.617
	Both OW and minimal breast growth	0.320	0.038	2.730	0.298
Class 3	OW only	3.207	1.084	9.490	0.035

	Minimal breast growth only	9.853	2.904	33.431	0.000
	Both OW and minimal breast growth	9.440	2.820	31.603	0.000
Class 4	OW only	0.869	0.455	1.657	0.669
	Minimal breast growth only	0.837	0.302	2.315	0.732
	Both OW and minimal breast growth	0.608	0.210	1.764	0.360
Class 2	OW only	0.139	0.029	0.663	0.013
	Advanced maternal age only	0.824	0.227	2.987	0.768
	Both OW and advanced maternal age	0.302	0.057	1.611	0.161
Class 3	OW only	1.332	0.510	3.479	0.558
	Advanced maternal age only	1.324	0.397	4.418	0.648
	Both OW and advanced maternal age	3.728	1.319	10.538	0.013
Class 4	OW only	0.883	0.449	1.736	0.718
	Advanced maternal age only	0.535	0.219	1.312	0.172
	Both OW and advanced maternal age	0.374	0.139	1.003	0.051
Class 2	GDM only	1.726	0.506	5.890	0.383
	Minimal breast growth only	1.883	0.452	7.841	0.384
	Both GDM and minimal breast growth	1.049	0.116	9.526	0.966
Class 3	GDM only	4.993	2.112	11.807	<0.001
	Minimal breast growth only	0.997	0.326	3.049	0.995
	Both GDM and minimal breast growth	3.841	1.333	11.068	0.013
Class 4	GDM only	1.279	0.550	2.973	0.568
	Minimal breast growth only	2.923	1.421	6.013	0.004
	Both GDM and minimal breast growth	0.704	0.184	2.702	0.609
Class 2	GDM only	1.746	0.401	7.602	0.458
	Advanced maternal age only	1.262	0.377	4.220	0.705
	Both GDM and advanced maternal age	1.013	0.111	9.277	0.991
Class 3	GDM only	0.570	0.163	2.001	0.381
	Advanced maternal age only	1.637	0.690	3.884	0.263
	Both GDM and advanced maternal age	2.614	0.915	7.464	0.073
Class 4	GDM only	1.866	0.848	4.108	0.121
	Advanced maternal age only	0.435	0.192	0.988	0.047
	Both GDM and advanced maternal age	1.122	0.437	2.883	0.810
Class 2	Minimal breast growth only	1.564	0.453	5.404	0.479
	Advanced maternal age only	1.214	0.362	4.063	0.754
	Both minimal breast growth and advanced maternal age	0.984	0.100	9.670	0.989
Class 3	Minimal breast growth only	3.892	1.499	10.104	0.005
	Advanced maternal age only	1.760	0.700	4.424	0.229
	Both minimal breast growth and advanced maternal age	9.199	2.995	28.253	<0.001
Class 4	Minimal breast growth only	1.106	0.492	2.486	0.807
	Advanced maternal age only	0.614	0.306	1.232	0.170
	Both minimal breast growth and advanced maternal age	0.092	0.011	0.775	0.028

CI: confidence interval; GDM: gestational diabetes mellitus; OR: odds ratio; OW: overweight/obese weight.
